# Anatomical Assessment vs. Pullback REsting full-cycle rAtio (RFR) Measurement for Evaluation of Focal and Diffuse CoronarY Disease: Rationale and Design of the “READY Register”

**DOI:** 10.3389/fcvm.2021.784220

**Published:** 2021-12-13

**Authors:** Zsolt Kőszegi, Balázs Berta, Gábor G. Tóth, Balázs Tar, Áron Üveges, András Ágoston, Attila Szücs, Gábor Tamás Szabó, Judit Barta, Tibor Szük, Dániel Czuriga, András Komócsi, Zoltán Ruzsa

**Affiliations:** ^1^Szabolcs—Szatmár—Bereg County Hospitals, University Teaching Hospital, Nyíregyháza, Hungary; ^2^Kálmán Laki Doctoral School of Biomedical and Clinical Sciences, University of Debrecen, Debrecen, Hungary; ^3^Department of Cardiology, Faculty of Medicine, University of Debrecen, Debrecen, Hungary; ^4^Invasive Cardiology Department, Bács-Kiskun County Hospital Kecskemet, Kecskemét, Hungary; ^5^Division of Cardiology, University Heart Center Graz, Medical University Graz, Graz, Austria; ^6^Heart Institute, Medical School of University of Pécs, Pécs, Hungary; ^7^Cardiology Center, Invasive Cardiology Unit, University of Szeged, Szeged, Hungary

**Keywords:** resting full-cycle ratio (RFR), microvascular coronary disease, fractional flow reserve (FFR), coronary flow reserve (CFR), coronary artery disease

## Abstract

**Background:** The morphology and functional severity of coronary stenosis show poor correlation. However, in clinical practice, the visual assessment of the invasive coronary angiography is still the most common means for evaluating coronary disease. The fractional flow reserve (FFR), the coronary flow reserve (CFR), and the resting full-cycle ratio (RFR) are established indices to determine the hemodynamic significance of a coronary stenosis.

**Design/Methods:** The READY register (NCT04857762) is a prospective, multicentre register of patients who underwent invasive intracoronary FFR and RFR measurement. The main aim of the registry is to compare the visual estimate of coronary lesions and the functional severity of the stenosis assessed by FFR, as well as the RFR pullback. Characterizations of the coronary vessel for predominantly focal, diffuse, or mixed type disease according to visual vs. RFR pullback determination will be compared. The secondary endpoint of the study is a composite of major adverse cardiac events, including death, myocardial infarction, and repeat coronary revascularization at 1 year. These endpoints will be compared in patients with non-ischemic FFR in the subgroup of cases where the local pressure drop indicates a focal lesion according to the definition of ΔRFR > 0.05 (for <25 mm segment length) and in the subgroup without significant ΔRFR. In case of an FFR value above 0.80, an extended physiological analysis is planned to diagnose or exclude microvascular disease using the CFR/FFR index. This includes novel flow dynamic modeling for CFR calculation (CFR_p−3D_).

**Conclusion:** The READY register will define the effect of RFR measurement on visual estimation-based clinical decision-making. It can identify a prognostic value of ΔRFR during RFR pullback, and it would also explore the frequency of microvascular disease in the patient population with FFR > 0.80.

**Clinical Trial Registration:** ClinicalTrials.gov (NCT04857762).

## Background

The functional assessment of coronary artery lesions plays an increasingly important role in the clinical decision-making process involving patients with chronic coronary syndromes (CCS). According to the current European Society of Cardiology (ESC) guideline on coronary revascularization, pressure-wire derived fractional flow reserve (FFR) measurement is recommended in patients with a 40–90% diameter stenosis on visual angiographic estimation and without prior evidence of ischemia (1). The latest ESC guideline on CCS also suggests the use of non-hyperemic instantaneous wave-free ratio (iFR) as an alternative of FFR for cardiovascular risk stratification and for the indication of revascularization (2). The current American guideline includes the diastolic pressure ratio (dPR) and the resting full-cycle ratio (RFR) as further non-hyperemic indices, to aid clinical decisions (3). Data are still lacking regarding the relation of visual assessment and RFR measurements.

Non-hyperemic parameters have the potential for (co)localizing the pressure drop(s) related to significant coronary lesion(s) during a pullback measurement along a coronary artery without adenosine administration (4, 5). Advantages of the recently developed RFR include the detection of the highest resting differences between proximal and distal pressures during the cardiac cycle. In contrast to iFR, RFR determines the gradient not only in a specific phase of the diastole. This offers potential benefits especially at gradient detection in the right coronary artery (6–8).

In recent years, the importance of intracoronary pressure conditions along the epicardial vessels has been increasingly recognized.

The prognostic importance of the definition of focal pressure drop was highlighted in the EMERALD study (NCT02374775), where the authors defined a ΔFFR_CT_ cut-off of 0.06 providing the most valuable information for the prediction of acute coronary events. Possible explanation can be according to the authors, that the large pressure drop across the lesion causes large net force acting on the plaque, which may facilitate plaque rupture (9).

Such a prognostic pressure-drop cut-off value for invasive hyperemic or non-hyperemic pullback measurements has not yet been established. We defined the focal disease criterion as ΔRFR >0.05 for <25 mm segment length which is the same gradient for a 1 mm unit (>0.002/mm), similarly as it was defined for the iFR. We set out to test this threshold for prognostic value in our registry during the follow-up of patients with negative FFR but positive focal ΔRFR (4).

While the main aim of the READY registry is to characterize the effect of RFR measurement on the visual estimation-based clinical decision-making, it may also provide guidance in cases with a non-ischemic FFR but with focal pressure drop (ΔRFR > 0.05).

We plan to analyze the follow-up data at 1 year not only according to the pre-specified categories but also to determine potential alternative cut-off values using receiver-operator characteristic (ROC) analysis. In addition, it will also explore the frequency of microvascular disease in the patient population with FFR > 0.80.

## Study Objectives and Design

The “*Anatomical assessment vs. pullback REsting full-cycle rAtio measurement for evaluation of focal and Diffuse coronarY disease (READY)*” register is an investigator-initiated, prospective, and multicentre observational study. Three hundred patients with coronary stenoses between 40 and 90% in diameter and clinical indication of intracoronary physiological assessment are planned to be included.

The study is approved by the Hungarian Institution of Pharmacy and Nutrition (OGYÉI/61148/2018) and registered on the ClinicalTrials.gov (NCT04857762). All the included patients will sign an informed consent form to participate in the registry. Execution of the study is supported by an unrestricted grant provided by the PremierGMed Cardio Ltd.

### Inclusion Criteria

Written, informed consent from all patients should be obtained for the enrolment of their details into the database of the Registry for potential future analysis.

Patients with CCS, who require functional intracoronary assessment with pressure guidewires, as part of the clinical management of their condition, are eligible to enroll if the stenosis in one main coronary branch is assessed to be in the 40–90% range of diameter reduction on the invasive coronary angiography.

### Exclusion Criteria

Patients with acute coronary syndrome, left main disease, contraindication for adenosine, coronary artery bypass graft on the investigated vessel, severe renal insufficiency (estimated glomerular filtration rate <30 ml/min/1.73 m^2^), any medical comorbidity resulting in life expectancy <12 months are excluded.

### Angiographic Evaluation Protocol

**Figure 1:**
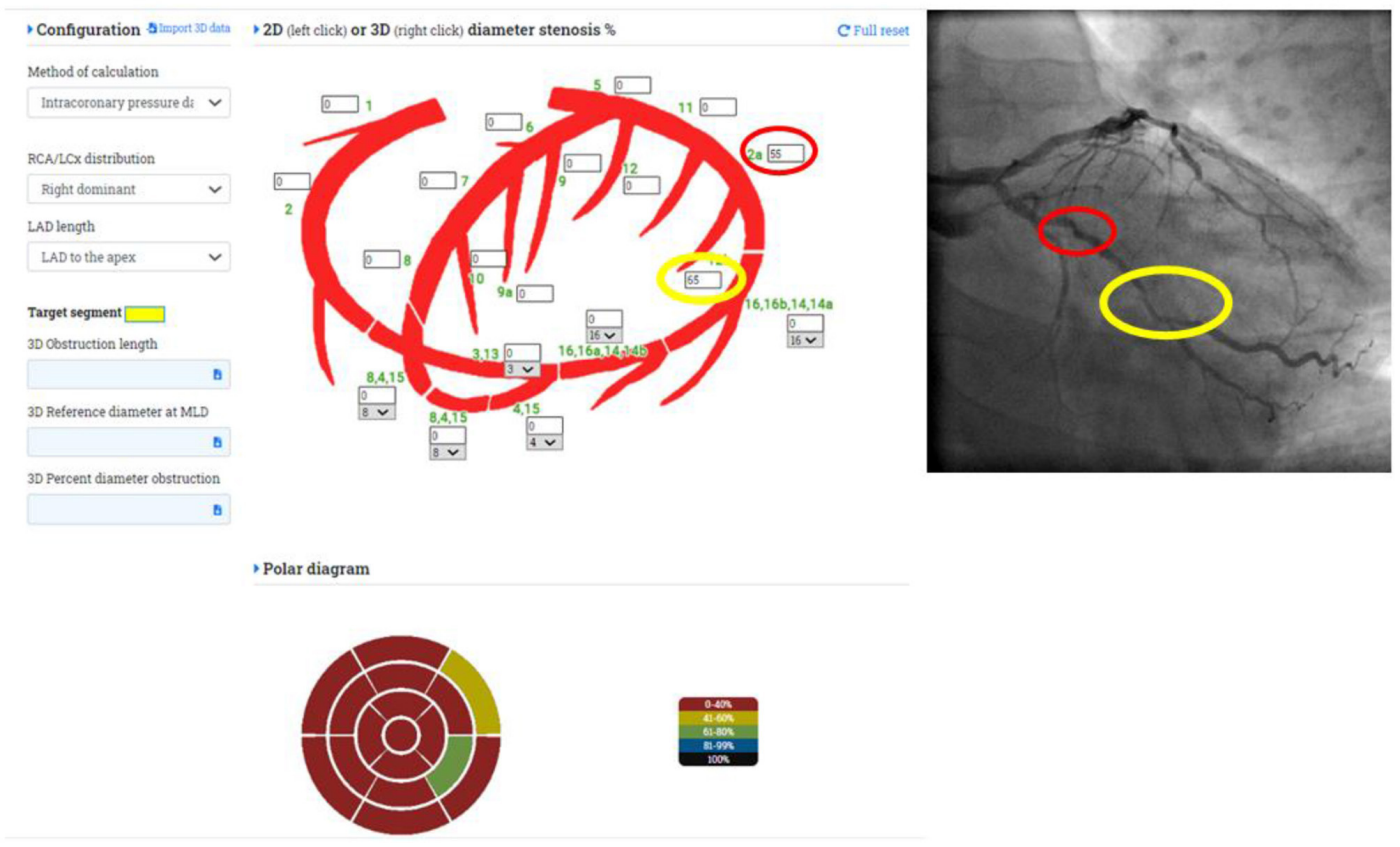
Definition of the investigated coronary segments and the corresponding left ventricular segments on the polar map.

Coronary diagnostic angiography is performed according to routine clinical practice. The visual estimate of the diameters stenosis % of the culprit lesion(s) should be included prospectively in the modified Syntax segmentation scheme (https://coronart.hu/), where the corresponding ventricular segments supply is indicated on a polar map (Figure 1). At the same time, the operator is required to record the characteristics of the coronary vessel disease as focal, diffuse, or mixed type, and to document their plan for angioplasty on the basis of the visual assessment.

### Invasive Coronary Physiology Assessment Protocol

PressurewireX will be advanced distally to the investigated lesion(s) of a coronary artery. Resting and hyperemic average pressures will be determined in this distal position (in FFR mode). Hyperemia will be induced by 200 μg intracoronary adenosine injection.

A resting manual pullback with 1–2 mm/s speed will be performed (in RFR mode) under simultaneous fluoroscopic control.

The lesion length can be measured exactly by 3D coronary reconstruction from two appropriate angiographic projections at least 25° apart. In case of incorporated 3D reconstruction software (e.g., PieMedical to the Canon or Siemens systems) this 3D measurement can be performed promptly. Alternatively, 2D measurement can be done from the best projection with minimal foreshortening.

**Figure 2:**
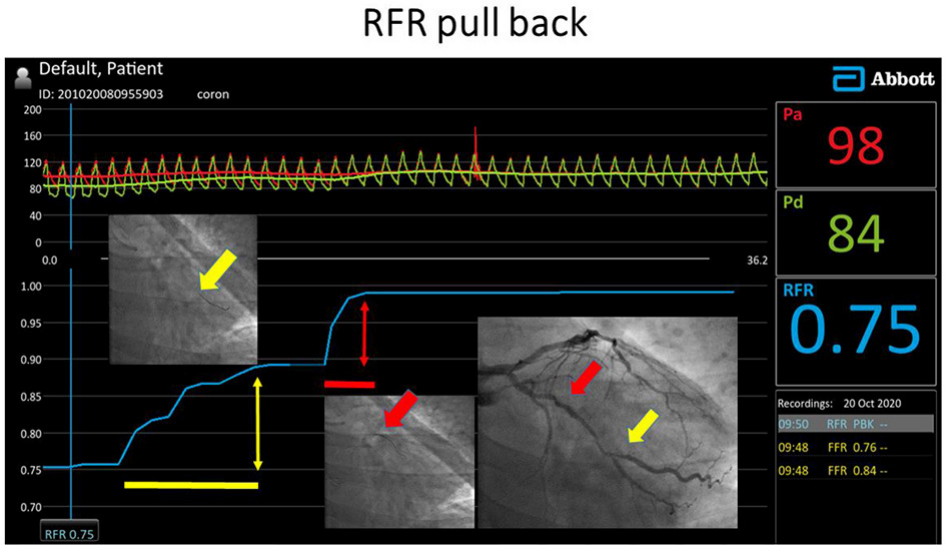
RFR pullback investigation in the left circumflex artery with intermediate stenoses. Both lesions proved to be significant according to the local ΔRFR.

The co-registration of the coronary angiography and the pressure pullback trace of the RFR measurement is based on the simultaneous fluoroscopic acquisition of the pressure wire position and the pressure trace. Using a reference image together to the pullback of the pressure wire under fluoroscopy can indicate quite precisely the beginning and the end of the lesion can be marked simultaneously on the Quantien equipment by the Marker function. The difference between the distal and the proximal value of the RFR markers will be used in the calculation of the Δ*RFR* (Figure 2).

I. The main aims of the READY register and its **primary endpoints:**

Our main aim is to compare the visual estimate of coronary lesions and the functional severity of the stenosis assessed by RFR pullback both on lesion and vessel levels.

Primary endpoint:

1. Characterization of the coronary vessel for predominantly focal/diffuse or mixed type disease according to visual vs. RFR pullback determination.

**Focal disease**: ΔRFR > 0.05 for <25 mm segment length (>0.002/mm)

**Diffuse disease**: ΔRFR > 0.05 for >25 mm segment length

If both the focal and diffuse criteria are fulfilled in the investigated vessel, then mixed type disease is diagnosed (Figure 2).

**Figure 3:**
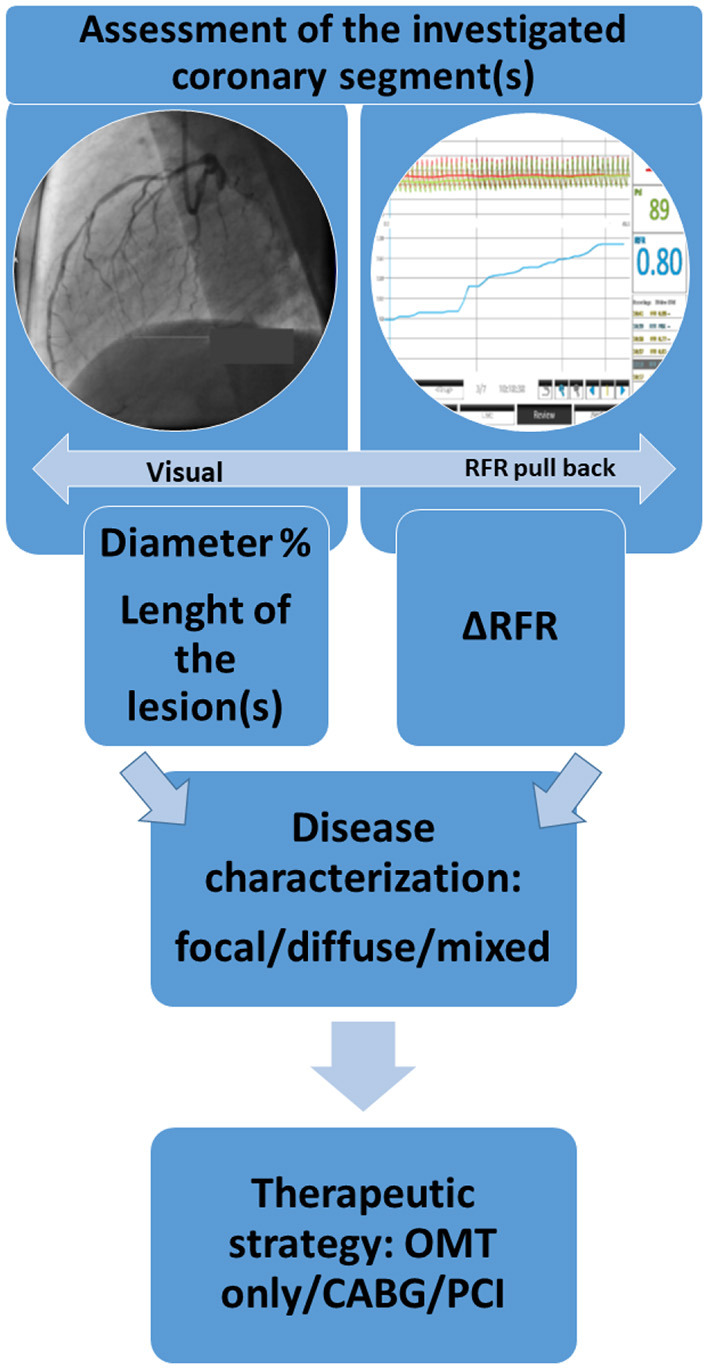
Flow chart of the comparison of the visual assessment and the results of the RFR measurement.

**II. Secondary endpoints:**
  1. The therapeutic strategy (conservative/PCI/CABG) will be compared based on visual evaluation vs. RFR measurements (Figure 3).

**Figure 4:**
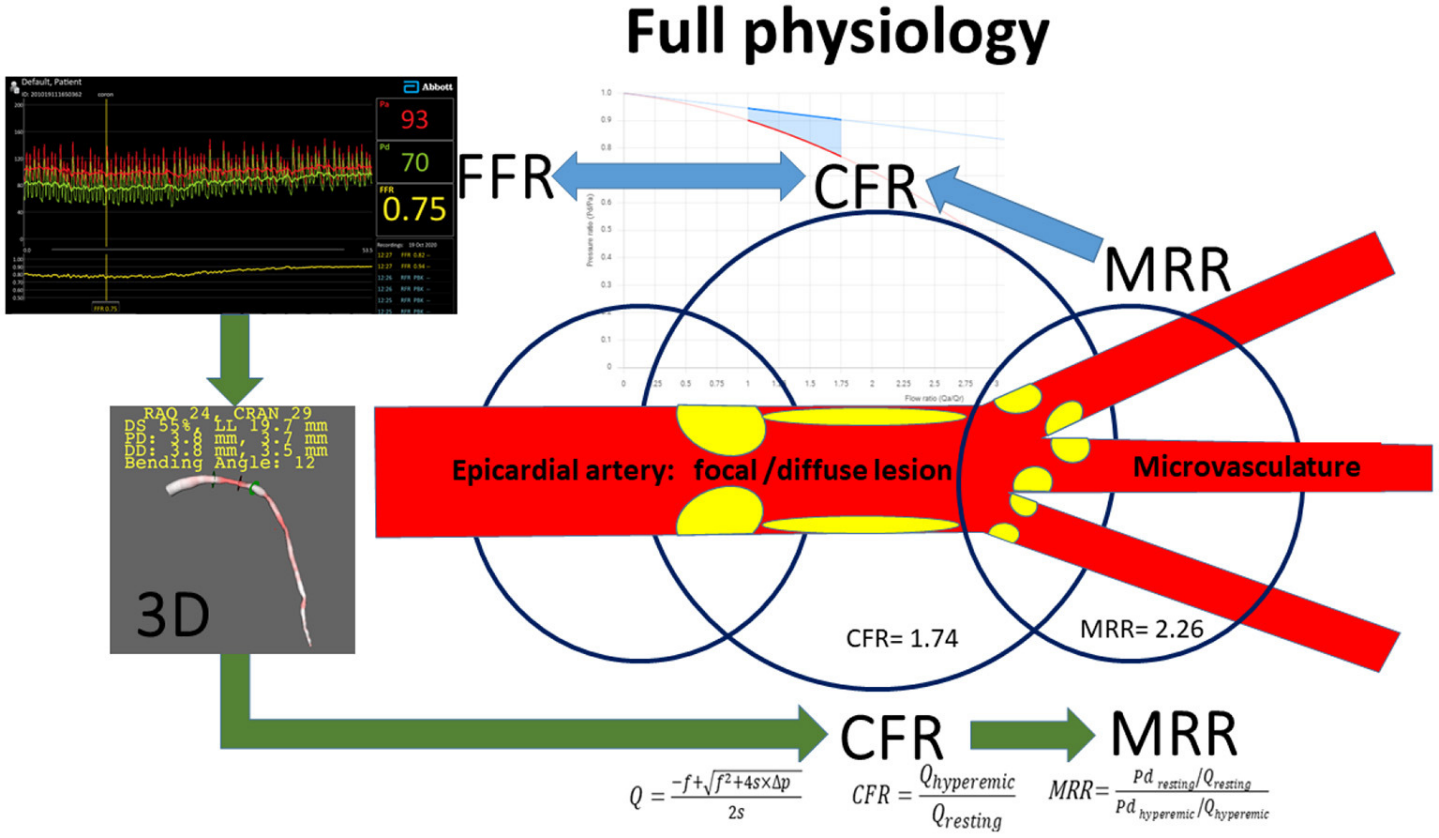
Summary of methods used in the extended physiology sub-study. RFR, resting full cycle ratio; FFR, fractional flow reserve; CFR, coronary flow reserve; MRR, microvascular resistance reserve; f, viscous friction losses; s, separation losses; Q, volumetric flow; ΔP, pressure gradient between the proximal and distal coronary pressure; Pd, distal coronary pressure.

Secondary endpoints in the subgroup of patients with negative FFR:

2. Secondary endpoint is defined only in the subgroup of patients with negative FFR as a composite of major adverse cardiac events, including death, type 1 myocardial infarction and coronary revascularization on the target vessel at 1 year. Type 1 myocardial infarction of the target vessel is defined according to the ESC classification (10). Revascularisation of the target vessel considered to be the composite secondary endpoint during the follow up.
3. Clinical endpoints will be compared in patients with non-ischemic FFR and the subgroup of cases where the local pressure drop indicates a focal lesion according to the definition of ΔRFR > 0.05 (for <25 mm segment length), and the subgroup without significant ΔRFR.
4. In the extended physiological sub-study, in cases where information regarding the microvascular state is desirable (i.e., patients with non-pathological FFR), the intracoronary average pressure values and the data of the 3D coronary reconstruction will be used for the calculations of coronary flow reserve (CFR) and the microvascular resistance reserve (MRR) (11–14). The rate of microvascular dysfunction will be determined in the investigated patient population (Figure 4).

A substudy of the register is planned to achieve extended physiological assessment in patients with the suspicion of microvascular disease in line with the latest ESC guideline stressing the role of microcirculatory dysfunction in the adverse outcome events in patients with non-significant coronary stenoses by FFR (class IIa, level B recommendation) (2).

In the extended physiological assessment, 3 parameters are calculated:

- Pressure bounded CFR interval (pbCFR)

According to the intracoronary average pressure values, the pbCFR interval will be calculated with the formula (11):


$$\begin{array}{l} \sqrt{\frac{Hyperemic\text{ Δ}p\;}{Resting\text{ Δ}p}}\leq pbCFR\leq \frac{Hyperemic\text{ Δ}p}{Resting\text{ Δ}p} \end{array}$$

If the cut-off value CFR = 2 lies inside the defined pbCFR interval, then a novel CFR calculation based on three-dimensional reconstruction and simple flow dynamic modeling will be performed to get the exact CFR_p−3D_ value to diagnose or exclude microvascular disease.

- Pressure and 3D derived CFR (CFR_p−3D_)

Hemodynamic calculations can be used that combines intracoronary pressure data and 3D anatomical parameters. The results of 3D angiographic reconstruction from the selected two angiograms and the hyperemic and resting pressure data are the basis of the simple hemodynamic equations calculating the ratio of the hyperemic and resting flow (CFR_p−3D_) (13, 14).

- CFR_p−3D_/FFR index

One simple possibility for the characterization of the microvasculature is to define the CFR_p−3D_/FFR index. If this value is below 2, the result indicates impaired microvascular vasodilator capacity (12, 13).

- Microvascular resistance reserve (MRR)

For getting more precise microvascular parameters, flow modeling using the data of the 3D coronary reconstruction and the intracoronary pressure values provide the microvascular resistance reserve (MRR) as detailed in the Figure 4 (13).

Vasodilatation is induced by intracoronary adenosine injection to determine the FFR value (left upper panel). For calculating a specific CFR and MRR values (CFR_p−3D_ and MRR_p−3D_) hemodynamic calculations are used that combines intracoronary pressure data and 3D anatomical parameters (left lower panel). Offline 3D angiographic reconstruction will be performed from the selected two angiograms of good quality, with at least 25° difference in angle, using a dedicated software (e.g., QAngio XA Research Edition 1.0, Medis Specials bv, Leiden).

Based on the hyperemic and the resting pressure data, simple hemodynamic equations calculate the resting and hyperemic flow, the CFR and the MRR (right lower panel). The pressure-flow relation is displayed by our freely available software https://coronart.hu/ in patient-specific flow range (upper middle panel) (13, 14). The CFR _p−3D_/FFR index can be expected to be very close to the calculated MRR value (in this example: 2.30 vs. 2.26, respectively).

The green arrows indicate the flow-chart of the calculations, while the blue arrows show the effect of the different hemodynamical parameters.

### Statistical Analysis

Descriptive statistics are planned as mean and SD, median (interquartile range) of the measured RFR and FFR values. Categorical variables will be compared with the Pearson χ^2^ test for focal/ diffuse/mixed type categorizations of the coronary disease by visual and physiological evaluations.

Correlation among variables will be determined by calculating Spearman ρ correlation coefficient.

Kaplan-Meier curves will be used to graphically display adverse events by focal lesion (ΔRFR ≥ 0.05/ 25 mm) and without focal lesion (ΔRFR < 0.05/ 25 mm) arms in the cases with FFR > 0.80. We plan to use survival analytic techniques such as a log-rank test to estimate the difference between the 2 subgroups.

The required sample size for the statistically significant (*p* < 0.05) difference with 80% power of the Kaplan-Meier curves of adverse events by focal lesion (ΔRFR ≥ 0.05/ 25 mm) and without focal lesion arms are 425, assuming 10 and 2% event rate in the two subgroups, respectively.

ROC analysis will be used to determine an optimal clinical cut of value, of ΔRFR (together its sensitivity and specificity) to predict a secondary endpoint at 1 year.

All analyses will be performed by using the Medcalc statistical software (MedCalc Software Ltd, Belgium).

## Discussion

A 50% diameter reduction of a coronary artery was established as the threshold of flow limitation in an experimental model during hyperemia (15); however, the relation between coronary stenosis and the resulting resting pressure gradient has not been systematically evaluated in humans. In patients with coronary artery disease, a poor correlation has been demonstrated between the anatomic and functional severity of stenoses, with more dissociation in the presence of diffuse atherosclerosis and arterial remodeling (16). In the clinical practice, visual assessment is still the most common basis for anatomical evaluation, despite the fact that the reproducibility of quantitative coronary angiography (QCA) outperformed the visual estimation of coronary stenosis (17).

For the determination of the effect of focal stenosis during hyperemia, Collet et al. defined deteriorated local FFR based on the analysis of curve variations in segments without functional disease by selecting the 95th percentile of FFR curve variation; a drop >0.0015 mmHg/mm as a cut-off to minimize the effect of minor artifacts on this parameter (18).

In our opinion, this approach can be useful for the distinguishing local and diffuse forms of coronary artery disease, however, its practical clinical utilization is limited. Even the authors of this publication have admitted that the overall feasibility of pullback pressure gradient (PPG) index calculation derived from the motorized FFR pullback was only 63%. The necessity of prolonged adenosine infusion may provoke discomfort of the patient, while the use of a motorized pullback device could be cumbersome. The clinical utilization of PPG is currently being tested in the Pullback Pressure Gradient Global Registry (PPG Global) (NCT04789317).

Another group used the hyperemic pullback to detect native focal lesions within the 20 mm segment or the intra-stent gradient by the definition of an FFR increase of ≥0.05 on the pullback curve. However, in that study among patients undergoing successful PCI, a physiology-guided incremental optimization strategy failed to improve the proportion of patients with an optimal result of FFR ≥ 0.9 (19).

It is also important to note that for iFR pullback, the trans-stenotic pressure gradients of >0.03 in a <15 mm segment were specified as focal lesions in the DEFINE PCI study (NCT03084367) (4).

In our study, we defined the focal disease criterion as ΔRFR > 0.05 for <25 mm segment length, which is the same gradient for a 1 mm unit (>0.002/mm), as was used for the iFR. Nevertheless, we decided to work with a longer segment length, corresponding to current stent practices using deliberately longer stents.

The non-hyperemic determination of the resting pressure gradient by pullback measurement has also the advantage of simplifying the prediction of the stent implantation's effect. In contrast to the hyperemic FFR measurement, in resting condition there is no cross-talk between the physiological effect of the target lesion and the rest of the vessel. As the resting flow will not be changed after stent implantation in most cases, the effect of eliminating the specific resistance of the vessel can be predicted straightforwardly (5).

**Figure 5:**
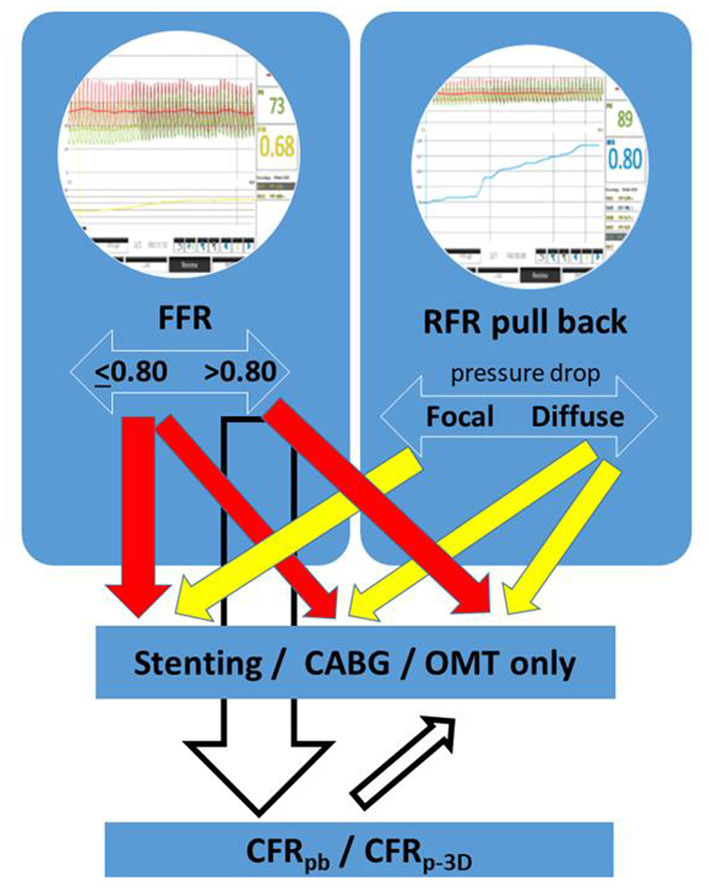
Flow-chart of the proposed clinical decision-making process according to the results of physiological measurements.

In this prospective registry, we aim to evaluate the additive value of intracoronary physiological measurements on clinical decision-making compared to visual estimation alone. We propose a flow-chart of the clinical decision-making process according to the results of physiological measurements (Figure 5). Hyperemic FFR measurement is intended to reflect the ischemia-inducing capability of the vessel. Furthermore, the characterization of the lesions to focal, diffuse or mixed types according to the RFR pullback has an important potential to influence the choice of treatment.

In the secondary endpoints of the register, we focused on the patient population with non-ischemic FFR value. During the 1-year follow-up, the prognostic value of the focal lesions (ΔRFR > 0.05) for cardiac adverse event will be investigated in this subgroup.

The characterization of the microvascular capacity may have also paramount relevance in cases of negative (>0.80) FFR values. If the extended physiological assessment shows low CFR/FFR index or MRR value, then microvascular disease is diagnosed with the impact on appropriate specific pharmaceutical treatment (20).

This relatively simple, combined resting and hyperemic assessment can provide a comprehensive physiological evaluation of the coronary disease with important treatment consequences.

## Data Availability Statement

The raw data supporting the conclusions of this article will be made available by the authors, without undue reservation.

## Ethics Statement

The studies involving human participants were reviewed and approved by University of Debrecen. The patients/participants provided their written informed consent to participate in this study.

## Author Contributions

ZK, ÁÜ, GT, AK, and ZR contributed to conception and design of the study. BT and AÁ organized the database. ZK performed the statistical analysis and wrote the first draft of the manuscript. GS, DC, and GT wrote sections of the manuscript. All authors contributed to manuscript revision, read, and approved the submitted version.

## Conflict of Interest

The patent of the method detailed in the extended physiology sub-study has been issued by the European Patent Office (WO2019175612, applicant: University of Debrecen).

## Publisher's Note

All claims expressed in this article are solely those of the authors and do not necessarily represent those of their affiliated organizations, or those of the publisher, the editors and the reviewers. Any product that may be evaluated in this article, or claim that may be made by its manufacturer, is not guaranteed or endorsed by the publisher.
